# Pure traumatic upper cervical disc herniation causing spinal cord injury: a case report and review of literature

**Published:** 2012-11

**Authors:** Guive Sharifi, Seyed Ali Mosavi, Misagh Shafieezad, Massoud Asgari Nosari

**Affiliations:** ^*a*^Neurosurgery Department, Loghman Hakim Hospital, Shaheed Beheshti University of Medical Sciences, Tehran, Iran.

## Abstract

One third of all spinal injuries involve cervical vertebrae, and the impact of injury to the cervical spinal cord is profound and requires systemic treatment.

The role and timing of surgical decompression after an acute spinal cord injury (SCI) remains one of the most controversial topics pertaining to spinal surgery. Lack of controlled, prospective, multicenter clinical studies has contributed to confusion in optimal treatment methods for patients with injuries of the cervical spinal cord. Clinically, the question of whether surgical decompression improves motor recovery following SCI remains surrounded by controversy.

**Case:**

Two cases with a 32-year-old man who developed right -sided Brown-Séquard Syndrome Following a motor to car accident with the large right paramedian C3–C4 disc herniation, and ipsilateral spinal cord compression and a 30-year-old man with the syndrome of acute central cervical spinal cord injury with motor impairment involving only upper extremities due to central C2–C3 disc herniation following a pedestrian accident are reported. Discectomy and anterior cervical fusion with the polyetheretherketone (PEEK) cage were performed. A complete motor deficit recovery and a marked sensitive deficit improvement were obtained.

The need for and timing of surgical decompression in post traumatic spinal cord injury is controversial. Surgery may expedite neurological recovery in some patients and may provide additional neurological recovery when clinical improvement has plateaued or worsened. In our patient a complete motor deficit recovery was observed.

**Keywords:**

Cervical, Disc herniation, Spinal cord injury


					Volume 4, Suppl. 1 Nov 2012
Publisher: Kermanshah University of Medical Sciences
URL: http://www.jivresearch.org
Copyright:
Congress of Iranian Neurosurgeons, 14-16 November 2012, Kermanshah, Iran
Paper No. 67
Pure traumatic upper cervical disc herniation causing spinal
cord injury: a case report and review of literature
Guive Sharifi a
, Seyed Ali Mosavi a
, Misagh Shafieezad a,*
, Massoud Asgari Nosari a
a
Neurosurgery Department, Loghman Hakim Hospital, Shaheed Beheshti University of Medical Sciences, Tehran, Iran.
Abstract:
One third of all spinal injuries involve cervical vertebrae, and the impact of injury to the cervical spinal cord is
profound and requires systemic treatment.
The role and timing of surgical decompression after an acute spinal cord injury (SCI) remains one of the most
controversial topics pertaining to spinal surgery. Lack of controlled, prospective, multicenter clinical studies has
contributed to confusion in optimal treatment methods for patients with injuries of the cervical spinal cord. Clinically,
the question of whether surgical decompression improves motor recovery following SCI remains surrounded by
controversy.
Case: Two cases with a 32-year-old man who developed right -sided Brown-Sequard Syndrome Following a motor
to car accident with the large right paramedian C3-C4 disc herniation, and ipsilateral spinal cord compression and a
30-year-old man with the syndrome of acute central cervical spinal cord injury with motor impairment involving only
upper extremities due to central C2-C3 disc herniation following a pedestrian accident are reported. Discectomy
and anterior cervical fusion with the polyetheretherketone (PEEK) cage were performed. A complete motor deficit
recovery and a marked sensitive deficit improvement were obtained.
The need for and timing of surgical decompression in post traumatic spinal cord injury is controversial. Surgery may
expedite neurological recovery in some patients and may provide additional neurological recovery when clinical
improvement has plateaued or worsened. In our patient a complete motor deficit recovery was observed.
Keywords:
Cervical, Disc herniation, Spinal cord injury
* Corresponding Author at:
Misagh Shafizad: Neurosurgery Department, Loghman Hakim Hospital, Shaheed Beheshti University of Medical Sciences, Tehran, Iran.
Tel: 09111540432, Email: mishafizad@gmail.com, (Shafizad M.).

				

